# Proposal of Classification Criteria for HTLV-1-Associated Myelopathy/Tropical Spastic Paraparesis Disease Activity

**DOI:** 10.3389/fmicb.2018.01651

**Published:** 2018-07-25

**Authors:** Tomoo Sato, Naoko Yagishita, Keiko Tamaki, Eisuke Inoue, Daisuke Hasegawa, Misako Nagasaka, Hiroko Suzuki, Natsumi Araya, Ariella Coler-Reilly, Yasuhiro Hasegawa, Yoshio Tsuboi, Ayako Takata, Yoshihisa Yamano

**Affiliations:** ^1^Department of Rare Diseases Research, Institute of Medical Science, St. Marianna University School of Medicine, Kawasaki, Japan; ^2^Department of Neurology, Fukuoka University, Fukuoka, Japan; ^3^Medical Informatics, St. Marianna University School of Medicine, Kawasaki, Japan; ^4^Department of Oncology, Karmanos Cancer Institute, Wayne State University, Detroit, MI, United States; ^5^Department of Advanced Medical Innovation, St. Marianna University Graduate School of Medicine, Kawasaki, Japan; ^6^Department of Neurology, St. Marianna University School of Medicine, Kawasaki, Japan; ^7^Department of Preventive Medicine, St. Marianna University School of Medicine, Kawasaki, Japan

**Keywords:** HTLV-1, HAM/TSP, classification criteria, disease activity, biomarker, neopterin, CXCL10

## Abstract

Human T-lymphotropic virus type 1 (HTLV-1)-associated myelopathy/tropical spastic paraparesis (HAM/TSP) is a rare chronic neuroinflammatory disease. While the disease usually progresses slowly without remission, there is a subgroup of patients with rapid progression and another subgroup with very slow progression. However, there have been no reports to date that have successfully determined the criteria to differentiate these subgroups. Therefore, we initially conducted a statistical modeling analysis to explore representative patterns of disease progression using data from our nationwide HAM/TSP patient registration system (“HAM-net”). The latent class mixed model analysis on the retrospective data (*n* = 205) of disease progression measured by the change in Osame Motor Disability Score from the onset of the disease to diagnosis demonstrated three representative progression patterns of HAM/TSP. Next, to test the effect of the progression rate at the initial phase of the disease on long-term prognosis, we divided 312 “HAM-net” registered patients into three groups (rapid, slow, and very slow progressors) based on the progression rate, then analyzed long-term functional prognosis of each group using the Kaplan–Meier method. Our data clearly demonstrated that the rapid progression at the early phase of the disease is an important poor prognostic factor. Moreover, to determine the biomarkers capable of discriminating the difference in disease activity, we compared the value of potential biomarkers of HAM/TSP among rapid (*n* = 15), slow (*n* = 74), very slow (*n* = 7), and controls (non-HAM/TSP patients, *n* = 18). The cerebrospinal fluid (CSF) levels of neopterin and C-X-C motif chemokine 10 (CXCL10) were the most valuable markers to discriminate among rapid, slow, and very slow progressors. To differentiate between rapid and slow progressors, the cut-off values of neopterin and CXCL10 were determined to be 44 pmol/mL and 4400 pg/mL, respectively. Furthermore, to differentiate between slow and very slow progressors, these values were determined to be 5.5 pmol/mL and 320 pg/mL, respectively. Notably, we found that CSF levels of these markers in very slow progressors were within the reference range. Thus, we propose a new classification criteria for disease activity of HAM/TSP that may contribute to improving the treatment algorithm for HAM/TSP.

## Introduction

Human T-lymphotropic virus type 1 (HTLV-1), the first human retrovirus discovered (Poiesz et al., 1980), causes a chronic neuroinflammatory disease termed HTLV-1-associated myelopathy/tropical spastic paraparesis (HAM/TSP) (Gessain et al., 1985; Osame et al., 1986). Infiltrated HTLV-1-infected cells cause chronic spinal inflammation and spinal cord tissue damage, which lead to HAM/TSP (Yamano and Sato, 2012; Bangham et al., 2015). In patients with HAM/TSP, the most frequent symptom is lower limb motor dysfunction, followed by bladder/bowel dysfunction and sensory disturbance (Osame, 1990; De Castro-Costa et al., 2006). Corticosteroids and interferon (IFN)-α are currently available for the treatment of HAM/TSP. However, there are no studies that support the need for stratified medicine based on disease activity, and there is no definite treatment algorithm for the use of therapeutic drugs (corticosteroids and IFN-α).

There exists a wide variation in the progression rates of HAM/TSP (Coler-Reilly et al., 2016), which suggests the need for stratified medicine. Some studies have reported a subgroup of patients with rapid progression and another subgroup with very slow progression. One example of rapid progressors has been reported in a Japanese study, in which 14 of 151 patients (9.3%) deteriorated more than three grades of a motor disability score within 2 years before initial examination (Nakagawa et al., 1995). A study from Peru demonstrates that 34 of 158 patients (21.5%) were rapid progressors who were unable to walk without two canes within 2 years of disease onset (Gotuzzo et al., 2004). A short communication from Brazil indicates that there were 7 of 88 patients (8%) with subacute progression, which is defined as the need to use a wheel chair during the first 2 years after the onset of symptoms (Lima et al., 2007). In an United Kingdom cohort, 3 of 48 patients (6%) were unable to walk within 2 years (Martin et al., 2010). A Martinique study also reports that a subgroup of patients have a faster rate of progression (Olindo et al., 2006). In the United Kingdom cohort, as an example of a subgroup of patients with very slow progression, 6 of 48 patients (12.5%) had little progression and deteriorated by only 0.3 s/10 m/year in a timed walk test (Martin et al., 2010). These studies put into context the wide variation in the clinical course of HAM/TSP patients and the need for a treatment algorithm to allow physicians to accurately determine disease activity and provide appropriate treatment. However, as mentioned above, the definitions representing the subgroups have differed from one researcher to another. There are no reports in which the clinical course from the disease onset was classified by subgroups defined by statistical methods.

In addition, because HAM/TSP progresses unremittingly, it is necessary to identify disease activity markers that can estimate the progression rate of HAM/TSP as early as possible. Several biomarker candidates for disease activity have already been identified. First, the level of proviral load in peripheral blood mononuclear cells (PBMCs) correlates with the progression of motor dysfunction and is associated with long-term prognosis (Matsuzaki et al., 2001; Olindo et al., 2005). Next, cell counts, anti-HTLV-1 antibody titer and protein levels in the cerebrospinal fluid (CSF) were elevated in rapid progressors, suggesting an association with progression of the disease (Matsuura et al., 2016). Furthermore, the CSF levels of C-X-C motif chemokine 10 (CXCL10) and neopterin, which are mainly produced in IFN-γ-stimulated astrocytes (Ando et al., 2013; de Paula Martins et al., 2018), strongly correlate with the rate of disease progression (Sato et al., 2013). However, there are no biomarker studies to date that have successfully determined the criteria for differentiating the subgroups with different disease activity.

In this study, we aimed to develop a new classification criteria for disease activity of HAM/TSP in order to provide appropriate treatment based on disease activity. First, we classified “clinical course from disease onset” of untreated HAM/TSP patients without any distribution assumption by applying a chronological change of motor disability to a mathematical model. Based on the results, we defined the criteria in which patients were divided into three groups. Next, we identified biomarkers and their cut-off values for discriminating the three groups. By summarizing the above results, we developed HAM/TSP classification criteria for disease activity based on clinical course and biomarkers.

## Materials and Methods

### Ethical Considerations

The study was approved by the St. Marianna University School of Medicine Bioethics Committee (clinical course analysis using HAM/TSP patient registry data: Approval ID No. 2044, biomarker analysis: No. 1646) and the Fukuoka University Faculty of Medicine Bioethics Committee (biomarker analysis: No. 14-2-08). Prior to the collection of blood or CSF samples, all participants provided written informed consent permitting the analysis of their samples for research purposes as part of their clinical care.

### Participants

**FIGURE 1:**
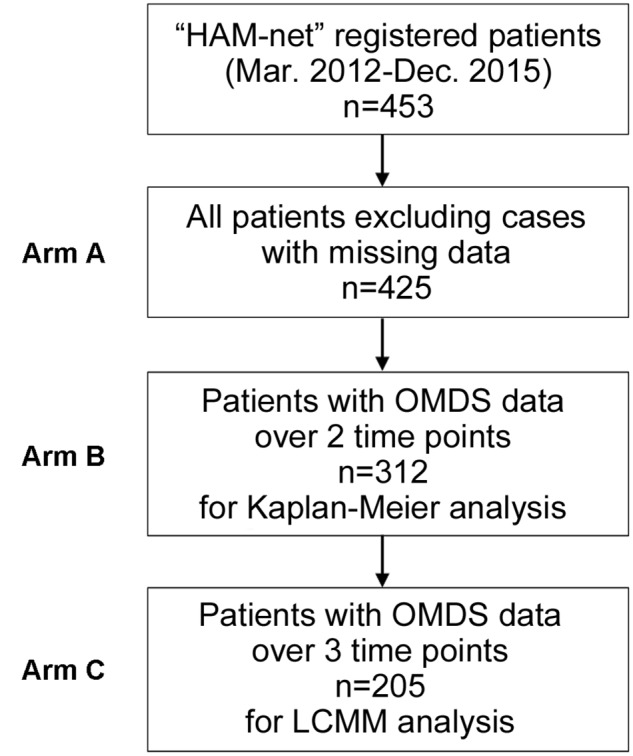
Patient flow chart. There were 453 HAM/TSP patients registered with the HAM/TSP patient registry “HAM-net” from March 2012 to December 2015. After excluding 28 cases missing survey data, the participants in this study were 425 patients (Arm A). Among them, 312 HAM/TSP patients (Arm B) who had at least 2 observations at different time points were included in the Kaplan–Meier analysis, and 205 patients (Arm C) who had at least 3 observations at different time points were included in the latent class mixed model (LCMM) analysis. OMDS, Osame motor disability score.

For clinical course analysis, retrospective data from patients who had enrolled in the Japanese HAM/TSP patient registry called “HAM-net” were used (Coler-Reilly et al., 2016). There were 453 patients registered with “HAM-net” from March 2012 until December 2015 (**Figure 1**). Survey data, including sex, age at onset, age at the onset of motor symptoms, age at diagnosis, age at the time of the survey, Osame motor disability score (OMDS) (**Table 1**), age at the time of deterioration in OMDS, medical treatment history, Health assessment questionnaire-disability index (HAQ-DI), family history of HAM/TSP and adult T-cell leukemia/lymphoma (ATL), and blood transfusion history were used for analysis. Among the 453 participants, 28 cases for which the above data were missing were excluded. Therefore, 425 cases were included in the analysis (Arm A shown in **Figure 1**) for preparing classification criteria based on clinical course from onset. Among these cases, 312 HAM/TSP patients (Arm B) who had at least 2 observations at different time points were applied to Kaplan–Meier analysis, and 205 patients (Arm C) who had at least 3 observations at different time points were applied to latent class mixed model (LCMM) analysis. As shown in **Table 2**, there were no statistical differences in age, sex, age at onset, or age at diagnosis among these three groups. However, there were significant differences in OMDS between Arm A and Arm C. The participants for biomarker analysis were 96 HAM/TSP patients with clinical course data and blood and CSF marker data obtained before treatment and 18 control cases [asymptomatic carriers (*n* = 10) and non-HTLV-1-infected non-inflammatory neurological disease patients (*n* = 8)].

**Table 1:** Osame motor disability score.

Grade	Motor disability
0	No walking or running abnormalities
1	Normal gait but runs slowly
2	Abnormal gait (stumbling, stiffness)
3	Unable to run
4	Needs handrail to climb stairs
5	Needs a cane (unilateral support) to walk
6	Needs bilateral support to walk
7	Can walk 5−10 m with bilateral support
8	Can walk 1−5 m with bilateral support
9	Cannot walk, but able to crawl
10	Cannot crawl, but able to move using arms
11	Cannot move around, but able to turn over in bed
12	Cannot turn over in bed
13	Cannot even move toes

**Table 2:** Demographics and clinical characteristics of “HAM-net”-registered HAM/TSP patients.

	Participants for LCMM analysis	Participants for Kaplan–Meier analysis	All registered patients	*p*-value
	*n* = 205	*n* = 312	*n* = 425	
Sex: Female	160 (78.0%)	240 (76.9%)	319 (75.1%)	N.S.^(a)^
Age at onset^∗^	41.7 ± 14.2	43.1 ± 14.3	44.5 ± 14.7	N.S.^(b)^
Age at diagnosis^∗^	52.1 ± 12.7	52.4 ± 12.7	52.3 ± 12.9	N.S.^(b)^
Age (at present^∗∗^)^∗^	62.3 ± 10.1	61.7 ± 10.5	61.9 ± 10.5	N.S.^(b)^
OMDS (range: 0−13)^∗^	6.1 ± 2.4^†^	6.4 ± 2.3	6.7 ± 2.3^†^	0.018^(b)^

*Statistical methods: (a) By Fisher’s exact test (b) by analysis of variance and Tukey post hoc tests.*

*^∗^Data are expressed as mean ± SD.*

*^∗∗^Present is defined as the time of the subject’s initial “HAM-net” interview.*

*^†^There is significant difference between OMDS in participants for latent class analysis and OMDS in all registered patients.*

*LCMM, latent class mixed model; OMDS, Osame motor disability score.*

### Measurement of Biomarkers

Peripheral blood mononuclear cells, serum and CSF samples were prepared as described previously (Sato et al., 2013). Briefly, PBMCs were isolated with standard procedures using Pancoll^®^ density gradient centrifugation (density 1.077 g/mL; PAN-Biotech GmbH, Aidenbach, Germany). Serum was obtained from venous blood samples by centrifugation after clotting. CSF was obtained by lumbar puncture. A small amount of CSF was used for routine laboratory tests, which included total protein, glucose, and cell counts. The remaining CSF was aliquoted into cryotubes and stored at -80°C until undergoing further analysis. The serum concentration of soluble IL-2 receptor (sIL-2R) was determined using a chemiluminescent enzyme immunoassay (LSI Medience Corporation, Tokyo, Japan). HTLV-1 proviral load was measured using real-time PCR, following DNA extraction from PBMCs, as previously described (Yamano et al., 2002). The anti-HTLV-1 antibody titer in CSF was determined using the gelatin particle agglutination test (Serodia-HTLV-1; Fujirebio, Tokyo, Japan). CSF neopterin level was measured using high-performance liquid chromatography at a commercial laboratory (SRL Inc., Tokyo, Japan). CXCL10 in CSF was measured using a cytometric bead array (BD Biosciences, Franklin Lakes, NJ, United States).

### Statistical Analysis

In order to explore representative patterns of disease progression for HAM/TSP patients, the LCMM was applied to longitudinally collected OMDS data. This method assumes the existence of latent class, which affects the longitudinal trajectory of an outcome variable under the given number of latent classes. Specifically, we used the cumulative distribution function of Beta distribution as a link function of this model, and 2–5 latent classes were specified. The goodness of fit of the model was evaluated by the Bayesian Information Criterion (BIC), and the model with the smallest BIC was the most optimal. The analysis set was data from HAM/TSP patients registered to “HAM-net” who had at least three observations at different time points (Arm C shown in **Figure 1**). To exclude treatment effects on OMDS, OMDS from onset to diagnosis were analyzed. Additionally, time to deteriorating to OMDS grade 6 for each group determined by LCMM analysis was evaluated using Kaplan–Meier method followed by a log-rank test. Median time to OMDS grade 6 was calculated for each group. Calculations were performed using R^1^ and the LCMM package^2^. Fisher’s exact tests were used for comparison of categorical variables. Analyses of variance and Tukey *post hoc* tests were used for comparison of continuous variables. Kruskal–Wallis tests followed by Dunn’s *post hoc* tests were used for comparison among the four groups for biomarker analysis. Jonckheere’s trend test was used to investigate whether each marker had an increasing trend from the control group to the rapid progressor group. Receiver operating characteristic (ROC) analysis was performed to examine the sensitivity and specificity of individual biomarkers. Optimal sensitivity and specificity are defined as those yielding the minimal value for (1 - sensitivity)^2^ + (1 - specificity)^2^. Statistical analyses and graph composition were performed using R, IBM SPSS Statistics Version 22 (IBM Corp. Armonk, NY, United States), or GraphPad Prism 6 (GraphPad Software, Inc., San Diego, CA, United States). All *p*-values were two-tailed, and the threshold of significance was set at 0.05.

## Results

### Classification of Clinical Course Patterns

**FIGURE 2:**
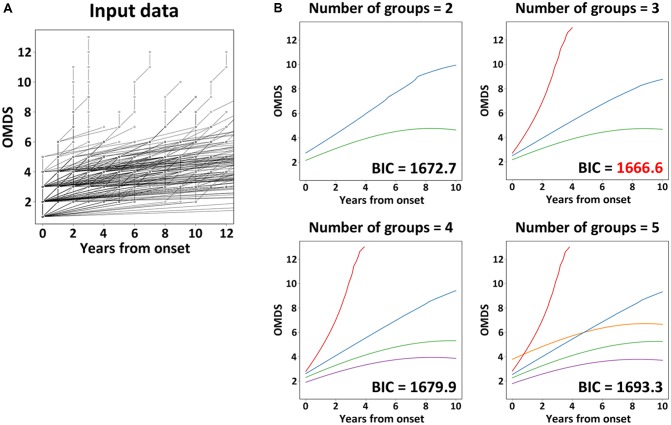
Latent class mixed model analysis. The graph **(A)** shows the chronological change of Osame motor disability score (OMDS) from the onset to diagnosis in patients with HAM/TSP (n = 205), which was used as input data for latent class mixed model (LCMM) analysis. The graphs **(B)** show the representative progression pattern of OMDS when we set 2–5 latent classes. The horizontal axis represents the elapsed years from onset. The vertical axis represents OMDS. BIC, Bayesian Information Criterion.

LCCM was utilized to classify the patterns of “clinical course from disease onset” of untreated HAM/TSP patients. The analysis set for LCMM analysis consisted of data from 205 HAM/TSP patients (Arm C shown in **Figure 1**). The scores of chronological change of motor disability of each patient were used as input data (**Figure 2A**) and applied to a mathematical model without any distribution assumption. As shown in **Figure 2B**, when hypothesizing the clinical course patterns to 2, 3, 4, or 5, the BIC were calculated as 1672.7, 1666.6, 1679.9, or 1693.3, respectively. As the reliability of the model correlates to lower BIC, the clinical course patterns subdivided to 3, was considered the most appropriate (right upper graph in **Figure 2B**). To include all patients who showed rapid progression, we defined rapid progressors as those who developed OMDS grade 5 or above within 2 years from the onset of motor symptoms. Determining those who show little or no signs of progression despite a lack of treatment (very slow progressors) is also clinically important, as this group of patients could avoid unnecessary treatment. When patients were divided into three groups, there were patients who would eventually progress to OMDS grade 5 or above (data not shown) even in the most indolent group (right upper graph in **Figure 2B**). Therefore, we referred to the slowest progression group when dividing patients into four groups (left lower graph in **Figure 2B**), and defined the very slow progressor group as those who were at or lower than OMDS grade 3 at 10 years from the onset of motor symptoms. Those who did not meet the criteria for either rapid progressors or very slow progressors were defined as slow progressors.

### Long-Term Functional Prognosis Based on the Three Groups

**FIGURE 3:**
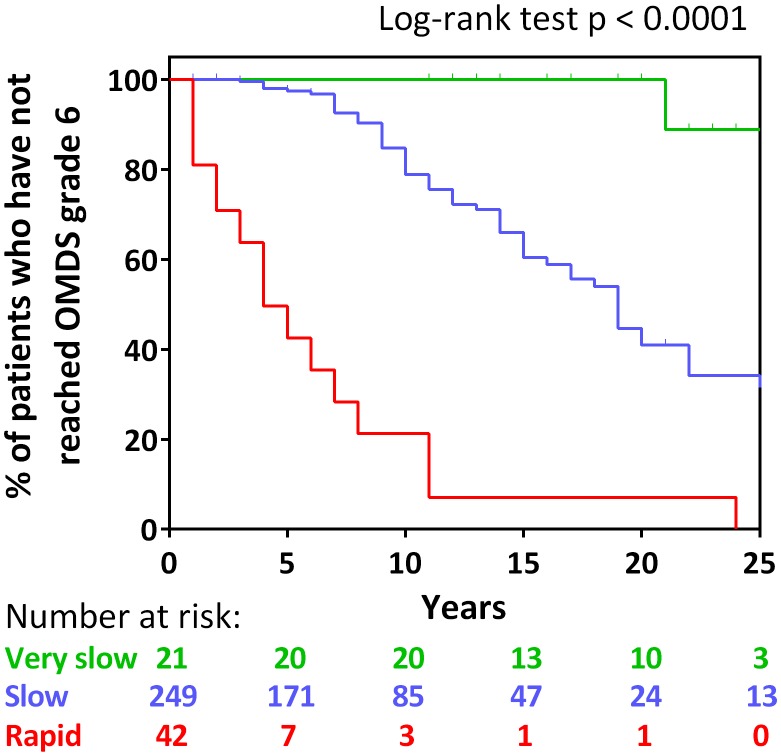
Kaplan–Meier analysis. Kaplan–Meier analysis to evaluate time from Osame motor disability score (OMDS) grade 2 to deteriorating to OMDS grade 6 for the following patient group defined by the difference of the clinical course from onset: rapid progressors (red line), slow progressors (blue line), and very slow progressors (green line). The horizontal axis represents the elapsed years from OMDS grade 2. The vertical axis represents percentage of patients who have not reached OMDS grade 6. Numbers shown below the graph indicate number at risk. Differences in the degree of progress among the three groups were tested by log-rank test. Significance was defined as p < 0.05.

To investigate whether the long-term prognoses of these three groups were different, Kaplan–Meier analysis was used to evaluate the time to progression from OMDS grade 2 to grade 6 in all three groups (**Figure 3**). The longitudinal data (including treatment period) on the progression of OMDS in HAM/TSP patients (*n* = 312; Arm B shown in **Figure 1**) were utilized in the analysis. When Arm B was divided into the three groups based on previously provided definitions, 42 (13.5%) were rapid progressors, 249 (79.8%) were slow progressors, and 21 (6.7%) were very slow progressors (**Figure 3**). The difference between the time to progress from OMDS grade 2 to grade 6 in these three groups (rapid, slow, and very slow progressors) was statistically significant among the three groups (*p* < 0.0001). Furthermore, the median time to progression from OMDS grade 2 to grade 6 was 4, 19, and 35 years, respectively. This shows that the classification criteria based on the “clinical course from disease onset” was successful in defining three groups with different prognoses.

### Characteristics of the Three Groups

**Table 3** shows the characteristics of all “HAM-net” registered patients divided into the three groups (*n* = 425; Arm A shown in **Figure 1**). Specifically, 15.5, 79.5, and 4.9% of the patients composed the rapid, slow, and very slow progressor groups, respectively. There were no differences in sex among the three groups, but there were statistically significant differences in age of onset, time to diagnosis, and duration of disease among the three groups (all *p* < 0.001). To be specific, the more rapid the rate of progression, the older the age at onset, and the shorter the time to diagnosis and duration of disease. Furthermore, there were differences in OMDS and HAQ-DI among the three groups (*p* = 0.003 and 0.001, respectively), with the rapid progressor groups demonstrating significantly higher scores in both OMDS and HAQ-DI than the other two groups. There were no significant differences among the three groups with regard to initial symptoms and family histories of HAM/TSP and ATL, but rapid progressors tended to carry less such family history. There were also no significant differences among the three groups with respect to transfusion histories, but the rapid progressors tended to have higher rates of transfusion. This result does not directly imply that there are a high number of patients with HAM/TSP attributable to blood transfusion in the rapid progressors group because the patients may have already been infected before transfusion or they may have been infected by horizontal transmission, independent of blood transfusion.

**Table 3:** Clinical attributes of rapid, slow, and very slow progressors who were defined based on the progression pattern after the onset of HAM/TSP.

	Group R Rapid progressor (*n* = 66, 15.5%)	Group SSlow progressor (*n* = 338, 79.5%)	Group VS Very slow progressor (*n* = 21, 4.9%)	*p*-value	Statistical methods	Groups with significant difference
Sex: Female	51 (77.3%)	255 (75.4%)	13 (61.9%)	N.S.	(a)	
Age at onset^∗^	55.9 ± 11.7	43.0 ± 14.0	32.4 ± 14.5	<0.001	(b)	R > S > VS
Age at diagnosis^∗^	58.7 ± 11.0	51.1 ± 12.9	51.0 ± 13.5	<0.001	(b)	R > S, R > VS
Age (at present^∗∗^)^∗^	65.6 ± 9.2	61.2 ± 10.6	62.1 ± 10.4	0.008	(b)	R > S
Diagnosis delay (time from onset to diagnosis)^∗^	2.7 ± 4.3	8.1 ± 8.2	18.6 ± 12.9	<0.001	(b)	R > S > VS
Disease duration (time from onset to present)^∗^	9.6 ± 7.7	18.2 ± 10.8	29.8 ± 10.0	<0.001	(b)	R > S > VS
**Initial symptoms (inclusive)**						
Gait disturbance	58 (87.9%)	277 (82.0%)	18 (85.7%)	N.S.	(a)	
Urinary disturbance	21 (31.8%)	145 (42.9%)	7 (33.3%)	N.S.	(a)	
Sensory disturbance (in legs)	10 (15.2%)	52 (15.4%)	2 (9.5%)	N.S.	(a)	
Others	17 (25.8%)	99 (29.3%)	7 (33.3%)	N.S.	(a)	
OMDS (range: 0−13)^∗^	6.6 ± 2.3	5.6 ± 2.2	5.4 ± 1.5	0.003	(b)	R > S, R > VS
HAQ-DI (range: 0−3)^∗^	1.4 ± 0.6	1.1 ± 0.7	1.0 ± 0.5	0.001	(b)	R > S, R > VS
Family history of HAM/TSP^†^: Yes	2 (3.0%)	36 (10.7%)	3 (14.3%)	N.S.	(a)	
Family history of ATL^‡^: Yes	2 (3.0%)	20 (5.9%)	1 (4.8%)	N.S.	(a)	
**History of blood transfusion**						
Yes (anytime)	19 (28.8%)	61 (18.0%)	2 (9.5%)	N.S.	(a)	
Yes, before 1986	16 (24.2%)	51 (15.1%)	2 (9.5%)	N.S.	(a)	

Statistical methods: (a) By Fisher’s exact test, (b) by analysis of variance and Tukey post hoc tests.

*Data are expressed as mean ± SD, **present is defined as the time of the subject’s initial “HAM-net” interview.

*^†^Family history of HAM/TSP indicates the subject has a first- or second-degree relative with HAM/TSP.*

*^‡^Family history of adult T-cell leukemia/lymphoma (ATL) indicates the subject has a first- or second degree relative with ATL.*

*N.S., not significant; OMDS, Osame motor disability score; HAQ-DI, health assessment questionnaire-disability index.*

### Biomarker Analyses

**FIGURE 4:**
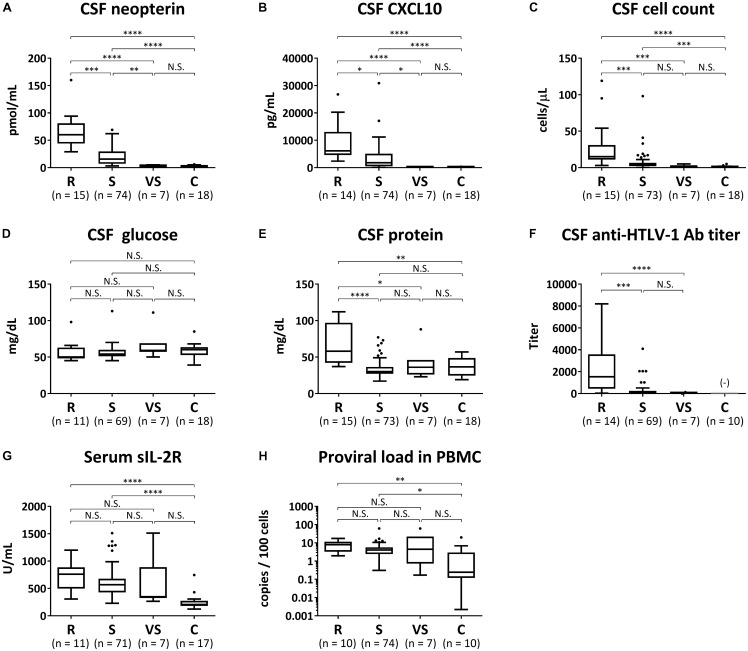
Biomarker analyses. The following eight candidate biomarkers were compared among rapid progressors (R), slow progressors (S), very slow progressors (VS), and controls (C): **(A)** cerebrospinal fluid (CSF) levels of neopterin, **(B)** C-X-C motif chemokine 10 (CXCL10), **(C)** cell count, **(D)** glucose, **(E)** total protein, and **(F)** anti-HTLV-1 antibody titer; **(G)** serum level of soluble IL-2 receptor (sIL-2R); **(H)** proviral loads in peripheral blood mononuclear cells (PBMC). Data are shown as a Tukey box plots: data are presented as median (interquartile range; IQR) and whiskers represent 1.5 IQR and black dots represent outliers. Statistical analysis was performed using the Kruskal–Wallis test followed by Dunn’s post hoc test: ^∗∗∗∗^p < 0.0001, ^∗∗∗^p < 0.001, ^∗∗^p < 0.01, ^∗^p < 0.05; N.S., not significant; Ab, antibody. (−) indicates that the CSF anti-HTLV-1-antibody test in normal control group was all negative.

Biomarker analyses were performed in 96 HAM/TSP cases in which blood and CSF marker data using pre-treatment samples were available and in 18 controls. The above 96 HAM/TSP patients were divided into the three patient groups (rapid, slow, and very slow progressors) defined by the difference of the clinical course from onset. Using a trend test, we investigated whether each of the eight candidate biomarkers had an increasing trend from the control group to the rapid progressor group (**Table 4**). All markers except CSF glucose had significantly increasing trends from the control group to the rapid progressor group. Next, we compared the eight candidate biomarkers among the three groups of HAM/TSP in addition to the control group (**Figure 4**). Consequently, CSF neopterin and CSF CXCL10 demonstrated significant differences in all pairwise comparisons among the three groups of rapid, slow, and very slow progressors (**Figures 4A,B**). The other six markers did not show statistically significant differences between slow and very slow progressors (**Figures 4C–H**). Notably, between very slow progressors and control participants, there were no statistically significant differences in any of the markers, with the exception of the CSF anti-HTLV-1 antibody titer. Regarding the antibody titer, all patients in the very slow progressor group tested positive for this antibody, whereas all 10 asymptomatic carriers in the control group tested negative (**Figure 4F**). As shown in **Table 5**, there were no differences among sex, but rapid progressors had a tendency to be older than the other groups and controls had a tendency to be younger than the other groups. Age of disease onset and OMDS were compared among the three disease groups. Age at onset was higher in the rapid progressors than in the other groups, and OMDS was progressively lower in the order of rapid, slow, and very slow progressors. This is similar to the results of the overall analysis of all “HAM-net”-registered patients shown in **Table 3** and provides a good representation of the patients.

**Table 4:** Trends for change in the values of candidate biomarkers from the control group to the rapid progressor group.

	Group R Rapid progressor^∗^		Group S Slow progressor^∗^		Group VS Very slow progressor^∗^		Group C Control^∗^		Jonckheere’s trend test *p*-value
CSF neopterin pmol/mL	60	(46.5−75.5)	15.5	(7−29)	4	(3−4)	3	(2−3)	<0.0001
CSF CXCL10 pg/mL	6128.5	(4836−12217)	1807.5	(543.8−5000.8)	246	(138−263.5)	138	(115−160.8)	<0.0001
CSF cell count cells/μL	15	(11.5−29)	4	(2−6)	1	(1−2.5)	1	(0.5−1)	<0.0001
CSF glucose mg/dL	50	(48−60)	54	(51−60)	59	(57−68.5)	60	(53.3−63)	0.9976
CSF protein mg/dL	58	(45−92)	30	(27−36)	36	(27.5−41)	36.5	(26.75−48)	0.0081
CSF anti-HTLV-1 Ab titer	1536	(512−2048)	64	(32−256)	32	(32−32)	(−)^∗∗^		<0.0001
Serum soluble IL-2R U/mL	761	(577.5−849)	568	(431−678)	349	(332.5−663)	208	(181−273)	<0.0001
Proviral load in PBMC copies/100 cells	7.95	(3.62−9.87)	4.04	(2.47−5.60)	4.44	(1.91−19.35)	0.25	(0.16−1.41)	0.0018

*^∗^Data is expressed as median (interquartile range). ^∗∗^(−) indicates that the CSF anti-HTLV-1-antibody test in the normal control group was all negative. CSF, cerebrospinal fluid; Ab, antibody; IL-2R, IL-2 receptor; PBMC, peripheral blood mononuclear cells.*

**Table 5:** Demographics and clinical characteristics of HAM/TSP patients for biomarker analysis.

	Group R Rapid progressor (*n* = 15)	Group S Slow progressor (*n* = 74)	Group VS Very slow progressor (*n* = 7)	Group C Control^∗^ (*n* = 18)	*p*-value	Statistical methods	Groups with significant difference
Sex: Female	12 (80.0%)	60 (81.1%)	3 (42.9%)	14 (77.8%)	0.1602	(a)	
Age (at present^†^)^∗∗^	67.0 ± 8.6	59.0 ± 10.3	59.1 ± 10.5	48.3 ± 13.1	<0.0001	(b)	R > S, R > C, S > C
Age at onset^∗∗^	63.9 ± 9.4	44.0 ± 13.7	45.0 ± 12.3	−	<0.0001	(b)	R > S, R > VS
OMDS (range: 0−13)^∗∗^	6.7 ± 1.9	5.4 ± 1.8	3.0 ± 0.8	−	<0.0001	(b)	R > S > VS

*Statistical methods: (a) By Fisher’s exact test, (b) by analysis of variance and Tukey post hoc tests.*

*^∗^Normal control group consists of healthy HTLV-1 carrier (n = 10) and non-HTLV-1-infected non-inflammatory neurological disease patients (n = 8), ^∗∗^Data are expressed as mean ± SD, ^†^Present is defined as the time of the sample collection.*

*OMDS, Osame Motor Disability Score.*

### Determination of Cut-Off Values

**FIGURE 5:**
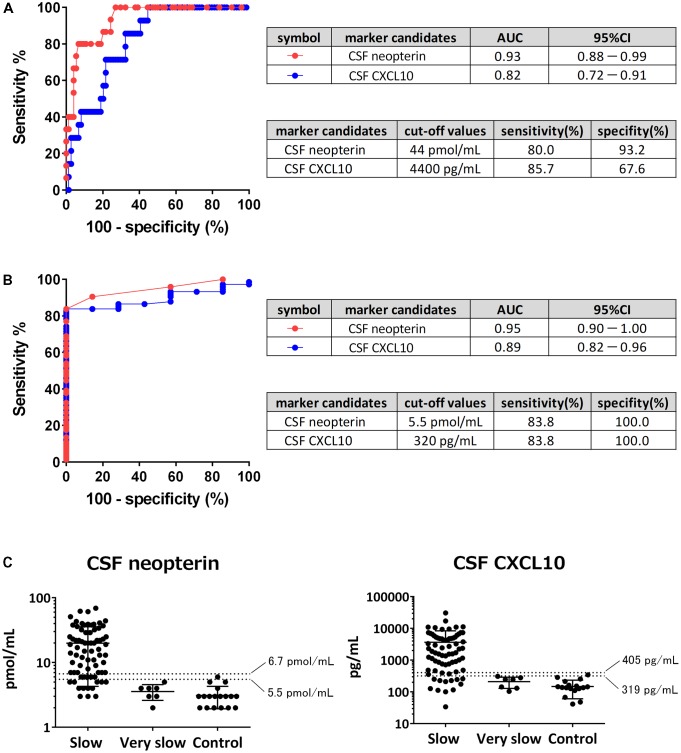
Determination of cut-off values. **(A)** Receiver operating characteristic (ROC) analysis was employed to evaluate the sensitivities and specificities of cerebrospinal fluid (CSF) neopterin and CSF CXCL10 for discriminating rapid progressors from slow progressors. Greater proximity of the ROC curve to the upper left corner indicates higher sensitivity and specificity of the marker. AUC, area under the ROC curve; 95% CI, 95% confidence interval. **(B)** ROC analysis was employed to evaluate the sensitivities and specificities of CSF neopterin and CSF CXCL10 for discriminating slow progressors from very slow progressors. **(C)** CSF levels of neopterin and CXCL10 were compared among slow progressors, very slow progressors, and controls. The data are plotted on a logarithmic axis. Middle horizontal bars show the arithmetic mean and the error bars show plus or minus one standard deviation (SD). Horizontal dashed lines indicate mean + 3SD (upper) and mean + 2SD (lower) as reference values of each marker.

Cerebrospinal fluid neopterin and CSF CXCL10 were both significantly different among the three groups. An ROC analysis was performed to evaluate the accuracy of the markers in differentiating rapid from slow progressors and to determine the cut-off values (**Figure 5A**). The areas under the curve (AUC) of CSF neopterin and CSF CXCL10 were 0.93 and 0.82, respectively. Both were higher than 0.8, therefore, demonstrating accuracy as markers to differentiate the two groups. The optimal cut-off value of CSF neopterin was 44 pmol/mL, which provided a sensitivity of 80.0% and specificity of 93.2% for the detection of rapid progressors. Similarly, the optimal cut-off for CSF CXCL10 was 4400 pg/mL with a sensitivity and specificity of 85.7 and 67.6%, respectively. Next, an ROC analysis to compare between slow and very slow progressors was performed (**Figure 5B**). The AUC were 0.95 and 0.89, respectively, again both higher than 0.8. The optimal cut-off for CSF neopterin was 5.5 pmol/mL with a sensitivity of 83.8% and specificity of 100% to detect slow progressors. Similarly, the optimal cut-off for CSF CXCL10 was 320 pg/mL, with sensitivity and specificity of 83.8 and 100%, respectively.

As neither marker has a pre-existing reference range, we attempted to determine a range using the values derived from controls. When the reference value was set at the mean + 3 standard deviations (SD), the upper limits of normal values of CSF neopterin and CSF CXCL10 were 6.7 pmol/mL and 405 pg/mL, respectively (**Figure 5C**). When these numbers were utilized as cut-off values to differentiate between slow and very slow progressors, CSF neopterin had a sensitivity of 77.0% and specificity of 100%, while CSF CXCL10 had a sensitivity of 79.7% and specificity of 100%. When the reference value was set at the mean + 2SD, the upper limits of normal values of CSF neopterin and CSF CXCL10 were determined to be at 5.5 pmol/mL and 319 pg/mL (≈320 pg/mL), consistent with previously derived cut-off value to distinguish between slow and very slow progressors. With the sensitivity of both markers at 83.8% and the specificity of them at 100%, the reference values set at mean + 2SD provided better sensitivity when compared to the reference value set at mean + 3SD. Therefore, we concluded that the reference value was best when set at the mean + 2SD, as fewer slow progressors would be included in the normal reference range.

## Discussion

In this study, we were able to classify HAM/TSP patients into three groups based on the disease activity, which is assessed by “progression rate of motor dysfunction after the onset of motor symptoms” and “the concentrations of neopterin and CXCL10 in CSF.” Consequently, we propose a novel classification criteria in which HAM/TSP patients were divided into the three groups (High, Moderate, and Low) of the disease activity (**Table 6**).

**Table 6:** Proposed classification criteria for disease activity of HAM/TSP based on clinical course and biomarkers^∗^.

Disease activity	Criteria based on clinical course after the onset of motor symptoms	Criteria based on the biomarkers	
		CSF neopterin (pmol/mL)	CSF CXCL10 (pg/mL)
High	**Rapid progressor:** Progression to OMDS grade 5 or greater within 2 years after the onset of motor symptoms	≥44	≥4400
Moderate	**Slow progressor:** Patients who does not meet the definition of either rapid- or very slow-progressor	6−43	320−4399
Low	**Very slow progressor:** Progression to OMDS grade 3 or less at least 10 years after the onset of motor symptoms	≤5	<320

*^∗^At the judgment of disease activity in individual patient, there is no need to meet all items, need to be judged comprehensively.*

The statistical pattern classification analysis using the chronological OMDS data from untreated 205 HAM/TSP patients demonstrated that the progression pattern after the onset of motor symptoms was divided into three patterns (**Figure 2**). This finding supported the existence of previously described rapid progressors (Nakagawa et al., 1995; Gotuzzo et al., 2004; Olindo et al., 2006; Lima et al., 2007; Martin et al., 2010) and very slow progressors (Martin et al., 2010). Importantly, the Kaplan–Meier analysis revealed that the long-term functional prognoses were different in the three groups (rapid, slow, and very slow) defined from results of pattern classification (**Figure 3**), suggesting that the clinical course at the early stage determines the subsequent prognosis. Rapid progressors had a significantly poorer prognosis than the other patients. This data clearly showed that the rapid progression at the early phase of the disease is an important poor prognostic factor in HAM/TSP patients. Such poor prognosis in rapid progressors might be caused by a delay in diagnosis, inappropriate treatment when diagnosed, ineffectiveness of treatment, and high disease activity. Therefore, it is imperative to survey the actual conditions at diagnosis and the treatment patterns for rapid progressors to determine methods to improve their prognosis.

This study revealed that there was a very slow progressor patient group among HAM/TSP patients. These patients were OMDS grade 3 or lower 10 years from the onset of motor symptoms, even in the absence of steroid treatment. This category contained patients with low disease activity whose CSF marker levels, which reflect spinal inflammation, were as low as those of the control group, even in the absence of treatment for HAM/TSP (**Figures 4, 5C**). They were also characterized by a younger age of onset than seen in the other patient groups (**Table 3**). Meanwhile, the rapid progressors were characterized by high levels of spinal inflammatory markers and old age at onset, consistent with previous reports (**Figure 4** and **Table 3**) (Nakagawa et al., 1995; Gotuzzo et al., 2004; Matsuura et al., 2016). Since there have been some case reports about the rapid progression of HAM/TSP due to organ transplantation (Gout et al., 1990; Toro et al., 2003), organ transplantation seems to be one of the background factors for the rapid progressors. In fact, of the 425 patients shown in **Table 3**, one with HAM/TSP that was caused by renal transplantation was a rapid progressor. In addition to transplantation, environmental factors (infection route, co-infection status), host factors (genetic factors such as HLA and gene mutation), and viral factors (viral gene expression and mutation) are candidate background factors that characterize the different disease activity of the three groups. In the future, we will clarify the importance of each factor.

There are various limitations to determine the disease activity based on the clinical course from onset. For example, there could be difficulties in judging the disease activity in patients with a short disease duration, or there may be a risk of overestimation or underestimation depending on the degree of the patients’ complaints. To overcome these limitations, we further developed a more objective biomarker-based classification criteria. To date, several biomarker candidates to evaluate disease activity have been reported (Matsuzaki et al., 2001; Olindo et al., 2005; Sato et al., 2013; Matsuura et al., 2016). In the present study, among the eight candidate markers, both CSF neopterin and CSF CXCL10 were identified as markers that clearly distinguish the three groups with different disease activities. Furthermore, the cut-off values dividing the three groups were determined. The levels of two blood-derived markers (soluble IL-2 receptor and PBMC HTLV-1 proviral load) were significantly higher in HAM/TSP patients with rapid or slow progression compared with asymptomatic carriers (**Figures 4G,H**), but they could not distinguish among the three patient groups with different disease activity. Thus, at the moment, CSF tests are essential to determine disease activity. In addition, it has been reported that the cell counts, protein levels, and anti-HTLV-1 antibody titer in the CSF were elevated in rapid progressors (Matsuura et al., 2016). These markers showed significantly higher values in rapid progressors than in slow progressors in the present study as well. However, they showed no significant differences between slow and very slow progressors. Therefore, these markers were insufficient for classification criteria for disease activity. To be specific, when using 5 cells/μL as the reference value for CSF cell counts measurable in general practice, the sensitivity to detect slow progressors was extremely low at 28.8% (data not shown). This indicates that although CSF cell counts are within normal range, most HAM/TSP patients are in an active and progressive phase. This should be noted when evaluating the condition of these patients.

PBMC HTLV-1 proviral load is elevated in patients with rapid progression (Matsuzaki et al., 2001; Olindo et al., 2005) and is weakly but significantly correlated with CSF levels of neopterin and CXCL10 (Sato et al., 2013). Therefore, HTLV-1 proviral load was a candidate marker to divide patients into three groups with different disease activity. In the present study, HTLV-1 proviral load tended to be elevated in rapid progressors (**Table 4**). However, there were no statistically significant differences in HTLV-1 proviral load among the three groups (**Figure 4H**). This may be due to the small sample size and the fact that the slow and very slow progressor groups included patients with a high proviral load (**Figure 4H**). Interestingly, the number of IFN-γ-producing infected T cells is more correlated with disease activity than with the proviral load itself (Yamano et al., 2009). Additionally, it has recently been found that a certain percentage of HAM/TSP patients have increased ATL-like infected cells (CADM1+CD7- CD4 T-cells) (MN et al. unpublished data). This evidence suggests that HTLV-1-infected clones that reside in one HAM/TSP patient do not necessarily consist only of pro-inflammatory clones that contribute to the disease activity of HAM/TSP. Thus, to evaluate the disease activity using the number of infected cells, methods for evaluating the amount of proinflammatory clones rather than the proviral load itself must be created.

The results of this study suggest that CSF levels of neopterin and CXCL10 are not only biomarkers for evaluating disease activity, but also may be candidate biomarkers for drug response or surrogate markers reflecting a long-term functional prognosis that should be the true endpoint of HAM/TSP. This is likely, given that corticosteroid therapy decreases CSF neopterin levels (Nakagawa et al., 1996; Nagai et al., 2013) and appears to decrease CSF CXCL10 levels as well (unpublished data). Because levels of these two markers reflect disease activity and are related to the progression rate of HAM/TSP, the decrease in their levels after treatment is expected to provide a decrease in the progression rate of HAM/TSP and to predict an improved prognosis. Interestingly, a multicenter retrospective cohort study indicates that oral low-dose corticosteroid therapy reduces the progression rate of HAM/TSP and improves the long-term prognosis compared to untreated patients (Coler-Reilly et al., 2017). Therefore, the levels of these two markers reflect the therapeutic effect and long-term prognosis of HAM/TSP, indicating their potential as drug response or surrogate markers. Moreover, CSF levels of neopterin and CXCL10 were within the normal range in HAM/TSP patients with low disease activity as based on our developed classification criteria. This suggests that the level of spinal cord inflammation is comparable to that of the control group. Oral corticosteroids can be used to reduce the level of inflammation in HAM/TSP patients with high or medium disease activity (Nagai et al., 2013). However, in patients with low disease activity where the inflammation level is in the normal range in the untreated state, oral corticosteroid therapy may have little benefit and may result in an increased risk of side effects by corticosteroids. Therefore, evaluation of the disease activity could give us important information in determining the need for corticosteroid therapy. Because it is critical to prospectively investigate these hypotheses, we have been conducting a confirmatory multi-center Phase 2b study (UMIN trial number, UMIN000023798) on the efficacy and safety of stratified corticosteroid therapy for HAM/TSP patients. This clinical trial should provide significant evidence to test these hypotheses.

This study has several limitations. The first limitation is that we could not adjust for potential confounders (age and OMDS) because of the small sample size of each group after the patients with HAM/TSP were divided into the three groups to examine biomarkers. In this regard, both CSF neopterin and CSF CXCL10 have a strong correlation with the progression rate of HAM/TSP after adjustment for OMDS (Sato et al., 2013). This finding indicates that both markers do not simply reflect the severity of motor disability. Although a difference in age between slow and very slow progressors was not observed in this study, the levels of both markers were significantly different, suggesting that the influence of age is limited. Therefore, it is possible that the change in the level of both markers reflects the disease activity rather than age or disease severity. The second limitation is that the cut-off values and reference values of the biomarkers determined in this study may vary from other studies, depending on measurement methods and experimental environments. Thus, it is essential to standardize the measurement methods of neopterin and CXCL10. Thereafter, it will be necessary to determine the cut-off values and the reference values again. The third limitation is that the classification criteria were determined based on retrospective data. Therefore, it is necessary to prospectively verify the validity of this classification criteria in the future.

## Conclusion

We herein propose a new classification criteria for disease activity of HAM/TSP. This classification criteria will enable clinicians to evaluate the disease activity early and support clinicians in providing appropriate treatment for each patient. This new classification criteria shall assist with establishing a novel therapeutic algorithm of HAM/TSP and incorporating tailored medicine based on the disease activity of each individual.

## Author Contributions

TS and YY contributed to the conception and design of the study. NY, EI, HS, AT, and YY run the patient registry “HAM-net.” TS, KT, YH, YT, and YY contributed to the biomarker analysis. TS and EI performed the statistical analysis. TS, DH, NA, AC-R, MN, and YY drafted and corrected the manuscript. All authors contributed to manuscript revision, read, and approved the submitted version.

## Conflict of Interest Statement

The authors declare that the research was conducted in the absence of any commercial or financial relationships that could be construed as a potential conflict of interest. The reviewer GT declared a past co-authorship with one of the authors YY to the handling Editor.

## Footnotes

1 http://cran.r-project.org

2 https://CRAN.R-project.org/package=lcmm
